# Assessing the size at maturity, spawning, and condition of the truncate soft-shell clam (*Mya truncata*) of southern Baffin Island, Nunavut, Canada

**DOI:** 10.7717/peerj.13231

**Published:** 2022-06-13

**Authors:** Jessica M. Wood, Meghan Donovan, Scott M. Grant

**Affiliations:** Fisheries and Marine Institute, Memorial University of Newfoundland, St. John’s, Newfoundland and Labrador, Canada

**Keywords:** Soft-shell clams, Life history, Condition, Arctic, Nunavut, Frobisher Bay, Hudson Strait, *Mya truncata*

## Abstract

The truncate soft-shell clam *Mya truncata* is an important source of country food for Inuit communities across the territory of Nunavut, Canada. *M. truncata* also plays an important role in marine ecosystems, yet there is little understanding of their life history and condition in Canadian Arctic waters. To provide a foundation on which aspects of the life history and condition of *M. truncata* of Baffin Island can be monitored in the future with a changing climate and fishery development, this study estimated size at maturity and provides insights into the spawning cycle and weight-length condition indices of clams from inner Frobisher Bay and the north shore of the Hudson Strait. Male and female *M. truncata* exhibited similar lengths at 50% attainment of sexual maturity, 31 mm and 32 mm shell length (SL), respectively. Most (77%) of the sexually mature *M. truncata* collected from inner Frobisher Bay in late August and 35% of clams collected from the Hudson Strait in early September were in the ripe stage of gonadal development. These results lead us to suggest a spring spawning season and that *M. truncata* invest in gonadal development for the next year’s spawning during the late summer-early autumn ice-free season while phytoplankton concentrations are high. Dry bodyweight-SL relationships were used to show that *M. truncata* condition can differ significantly over small and large spatial scales based on plotted 95% confidence intervals.

## Introduction

The Inuit of the Canadian Arctic have traditionally depended upon harvesting country food for nourishment (Chan et al., 2006; NCRI, 2005). A popular source of country food for communities across Nunavut is the truncate soft-shell clam, *Mya truncata*, and where high densities occur there is interest in commercial fishery development. Because the Arctic marine environment is increasingly affected by climate change (Hinzman et al., 2005), marine species, including *M. truncata*, may be impacted in ways that alter their physiology and subsequent life history, condition, and reproductive potential. For example, rising temperatures and earlier ice break-up may increase growth rates and advance spawning times (Philippart et al., 2003; Steeves et al., 2018). In addition, increases in freshwater runoff and reductions in the geographic extent and duration of ice cover in marine environments are expected to increase the ratio of phytoplankton to ice algae over the course of a year and alter phytoplankton species and size composition (Blais et al., 2017; Makela, Witte & Archambault, 2017; Park et al., 2015; Smyth, Tyrell & Tarrant, 2004). Changes in the attributes of planktonic algae with climate change could affect body condition, or the mass of an individual at a given length, as *M. truncata* depends on phytoplankton for nutrition (Makela, Witte & Archambault, 2017; Sun et al., 2007). Subsequently, changes in condition could affect life history traits, reproductive potential, and recruitment. In a rapidly changing Arctic environment, it is important to provide baseline information on population condition and life history traits of *M. truncata* that could be affected by both harvesting and climate change.

In North America, *M*.* truncata* is typically found in coastal intertidal and subtidal waters of the Arctic, with its southern boundary identified as Massachusetts Bay, Massachusetts, U.S.A.  (Lubinsky, 1980). In the Canadian Arctic, *M. truncata* occur in the intertidal and subtidal zones where density has been observed to increase with depth, with maximum densities found at 40m in some areas (Siferd, 2005). Studies of *Mya arenaria* show juvenile soft-shell clams tend to occupy the intertidal zone, while adults tend to concentrate in the lower intertidal and subtidal zones (Matthiessen, 1960; Powers et al., 2006). *M. truncata* is an integral part of the Arctic ecosystem, providing a means of energy transfer from primary producers to bearded seals and walrus, two of the primary consumers of bivalve molluscs in Canada’s Arctic and sources of country food for Inuit (Banfield & Brooks, 1977; Welch et al., 1992). In recreational fisheries in Canada’s Arctic, clams are dug at low tide to contribute to Inuit diets, and so are essential for transferring energy and nutrients from sea to land.

Despite its ecological importance, the life history and condition of *M. truncata* in the Canadian Arctic is poorly understood. To date, studies of *M. truncata* in North America have focused on aging and growth, developing methods to estimate abundance through siphon counts, bioaccumulation, inferring past pollutant fluctuations from fossils, predator induced siphon regeneration, presence of *Toxoplasma gondii*, incorporation of fatty acids from sea ice algae, and the significance of this species as a biomonitor of wastewater (Atwell, Hobson & Welch, 1998; Bourgoin & Risk, 1987; Siferd, 1997; Siferd, 2005; Siferd & Welch, 1992; Welch & Martin-Bergmann, 1990; Welch et al., 1992; Amiraux et al., 2021; Fung et al., 2021; Schaefer, Deslauriers & Jeffries, 2021). Studies of *M. truncata* in other parts of the world have assessed spawning season, responses to heat stress, molecular characterization, and fatty acid composition, among other topics (Amaro, Duineveld & Tyler, 2005; Birkely, Grahl-Nielsen & Gulliksen, 2003; Sleight et al., 2018; Sparagano et al., 2002). From sustainable fishery development, conservation, and environmental monitoring perspectives it is also important to establish size at attainment of sexual maturity, spawning events, and an effective method to evaluate and compare the condition of populations of *M. truncata* in the Canadian Arctic. 

Condition indices compare the weight of an individual at a given length to individuals of a similar length, where the individual with the higher weight has a higher condition index (Rennie & Verdon, 2008). One method to assess and compare condition of *M. truncata* is to develop a standard weight equation derived from length-weight data representing the entire geographic range of the species (Murphy, Willis & Springer, 1991; Blackwell, Brown & Willis, 2000; Gerow, Hubert & Anderson-Sprecher, 2004). However, when it comes to *M. truncata*, data required to develop suitable standard weight equations based on the geographic extent of the species is lacking. Alternatively, condition studies involving clams have used a ratio of body weight to shell length (Ellis et al., 2002; Pillay, Branch & Forbes, 2007) and relationships between body weight and length can also be used to compare condition between populations.

Length-based condition indices need to be free from length-related bias. Otherwise, a change in condition within or between populations could simply result from a change in average size of individuals in the population and thereby misrepresent condition (Gerow, Hubert & Anderson-Sprecher, 2004). Misrepresentation of population condition could mislead conservation studies and fisheries management decisions. A reliable method to assess condition of *M. truncata* populations will need to eliminate length bias to produce reliable results.

This study examines the sex and stage of gonadal maturity of male and female *M. truncata* from two sites within inner Frobisher Bay and two sites on the north shore of the Hudson Strait to establish size at sexual maturity and provide information on the reproductive cycle and spawning in Canadian Arctic waters. In addition, the body condition of *M. truncata* was examined to investigate whether in the absence of data required to develop a standard weight equation, a ratio of body weight to shell length or weight-length relationships are best suited for evaluating and comparing the condition of *M. truncata*.

## Materials & Methods

### Study sites

**Figure 1:**
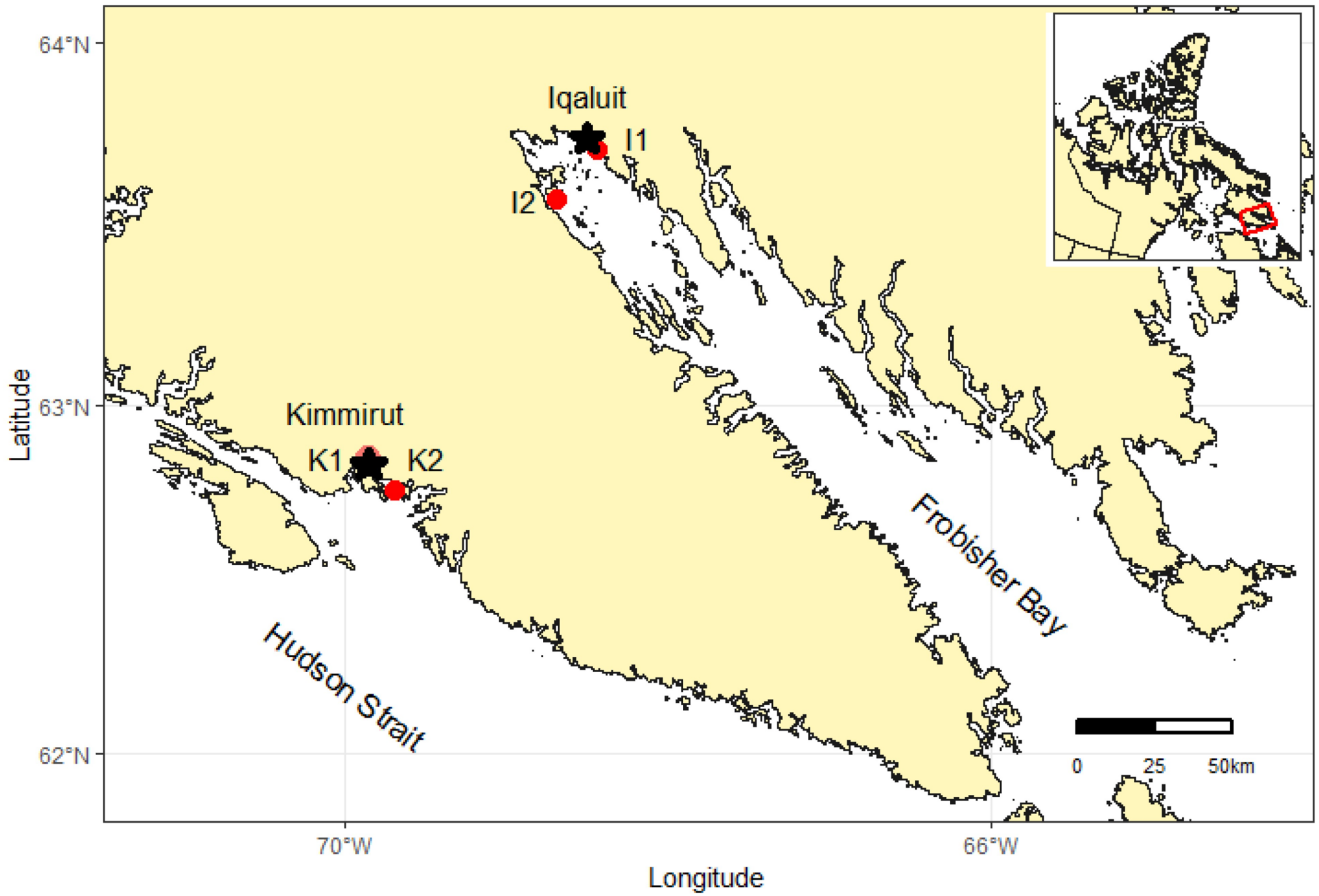
Map of southern Baffin Island showing the locations of two study communities (*i.e.,* Iqaluit and Kimmirut), and sites where *M. truncata* were collected near each community (I1, I2, K1, K2). Inset map illustrates the territory of Nunavut, Canada and study location. Map data from GADM database of Global Administrative Areas (http://gadm.org/).

Sampling sites were chosen through consultation with local Hunters and Trappers Associations to establish where communities collect clams during the ice-free season. *M. truncata* were collected at low tide from the intertidal zone at two sites within inner Frobisher Bay (hereafter FB), near the community of Iqaluit and two sites on the north shore of the Hudson Strait (hereafter HS) (*i.e.,* south coast of Baffin Island), near the community of Kimmirut (Fig. 1). One site near Iqaluit was accessible by road (Site I1), while the other was roughly 15 km across FB and only accessible by boat (Site I2). Similarly, one site near Kimmirut was accessible by road (Site K1), and the other was located roughly 15 km from the community into the Hudson Strait (Site K2). The substrate type of the intertidal zone at all sites was sand with sparsely distributed small and large rocks (10–30 cm diameter) often with attached fucoid macroalgae. The sediment type within Frobisher Bay has been further classified as sandy mud, with increasing gravelly sandy-mud with increased proximity to the community of Iqaluit (Deering et al., 2018). No published account of the sediment classification near Kimmirut was found, but the sediment at the study sites was observed to be similar to sites near Iqaluit. In FB, 99 clams were collected from 28–30 August 2018, with weather requiring three visits to collect clams from the two FB sites. In HS, 247 clams were collected from two sites on 12 September 2018. These sample dates coincided with the new moon phase when maximum tidal amplitudes of 10.4 m and 12.1 m occurred within Iqaluit and Kimmirut coastal waters, respectively. Clams were buried 10–30 cm in the sediment and were dug out from the lower intertidal zone using garden trowels. Community members indicated clams are concentrated in the lower intertidal zone to apparently avoid ice scour during the winter months. All clams encountered in the sediment were collected, with no discrimination for size. All clams were kept in cool sea water during transport back to the community where they were immediately frozen and maintained at −20 °C for subsequent analysis.

### Sex determination and gonadal staging

In the laboratory, frozen clams were thawed, and a Vernier caliper was used to measure shell length (SL; ±1.0 mm) from the anterior tip of the shell to the posterior edge. Clams were gently pried open, and a scalpel was used to sever the adductor muscles at the point of attachment to the shell. The entire animal, including the adductor muscles, was then removed from the shell. When present, a negligible sample (<0.001 g) of gonadal tissue was removed to determine sex and stage of gonadal development. Subsequently, the body of each clam, without the negligible sample of gonadal tissue, was individually dried to constant weight (±0.0001 g) at 60 °C. This analysis provided the dry body weight (DW) of each clam.

Clams that did not exhibit gonadal development (*i.e.,* no gonadal tissue visible on the clam’s body) could not be sexed and were classified as undifferentiated and considered immature. Gonadal smears were examined at 10–40 × magnification and used to assign each clam to one of five stages of gonadal development based on the scale defined by Brousseau (1987): (1) indifferent, (2) developing, (3) ripe, (4) spawning (partially spent), and (5) spent. To aid in classification, gonadal smears of *M. truncata* were also compared to photographs of histological preparations of gonadal tissue of *M. arenaria* from Ireland (Cross et al., 2012; Cross, 2011). In the current study, *M. truncata* classified as indifferent possessed gonadal follicles, but no lumen or cavity was present, and no developing egg or sperm cells were found in at least three gonadal smears examined for each clam. As such, clams classified as indifferent were considered to be immature and lumped with clams classified as undifferentiated. Clams with gonads that exhibited follicular lumen and possessed egg or sperm cells developing on the periphery of the lumen were classified as developing. Ripe clams displayed more fully developed egg or sperm cells with tails and many of these gametes were free within the follicular lumen. Although many of the egg and sperm cells had been discharged in clams classified as spawning, there were still some free gametes within the follicular lumen. In spent clams, the follicular lumen was empty and there were no developing eggs or sperm on the periphery of the lumen. None of the clams examined during this study were classified as spent.

### Maturity ogive

Separate male and female size at maturity ogives were constructed for clams collected across all sites by quantifying the proportion of male or female clams that were sexually mature within each five mm SL class. The sex ratio of sexually mature clams was used to estimate the sex of immature clams (*i.e.,* undifferentiated and indifferent stages combined). Specifically, the 1.14:1 female: male sex ratio was used wherein 53% of all immature clams within each five mm SL class were deemed female and 47% were deemed male. The best fit model for each maturity ogive was selected using AICc, where models tested included binomial (logit link and probit link), Gompertz, and Weibull distributions.

### Shell length analysis by site, sex, and maturity

A one-way analysis of variance (ANOVA) was used to examine variation in *M. truncata* SL among all four sample sites (*i.e.,* two in each of FB and HS). Tests of normality and equality of variance were performed using the Shapiro–Wilk’s normality test and the Levene median test, respectively. When assumptions of normality and equality of variance could not be met by transformation, the non-parametric Kruskal–Wallis ANOVA on ranks was used and Dunn’s post-hoc test if the result was significant. Tukey’s HSD post-hoc test was used when parametric analysis indicated significant differences.

A linear model was used to examine variation in SL among immature clams and mature male and mature female clams by community (*i.e.,* FB and HS) using the following equation:

(1) \begin{eqnarray*}{\mathrm{SL}}_{\mathrm{i}}={\mathrm{\beta }}_{0}+{\mathrm{\beta }}_{1}\mathrm{Community}\ast {\mathrm{\beta }}_{2}\mathrm{Sex}+.\end{eqnarray*}

Here, β_0_ represents the intercept, β_1_Community is the community where the clam was collected, either FB or HS, β_2_Sex is the sex of the clam, either immature, male, or female, and *ɛ* is the error term. Model assumptions were validated by testing for patterns in residuals of model covariates. Tukey’s HSD post-hoc test was performed on the model to make pairwise comparisons among SL at sex at each site.

### Length-based condition indices

A DW condition index was calculated for *M. truncata* using the following equation:

(2) \begin{eqnarray*}{\mathrm{CI}}_{\mathrm{DW}}=(\mathrm{DW}\div \mathrm{SL})\times 1000.\end{eqnarray*}

This equation multiplies (DW ÷ SL) by 1000 to avoid working with values <1. This dry weight condition index was used as opposed to wet weight indices as recommended by previous bivalve studies to avoid accounting for water content in condition calculations (Higgins, 1938; Mo & Nielson, 1992). As fish deplete their energy reserves, water content increases in muscle tissue and energy storage organs to replace lost protein and lipids, making dry weight condition indices the most accurate way to infer condition (Lucas & Beninger, 1985; Grant & Brown, 1999). Fulton’s condition factor (Ricker, 1975) was not used in this study because it is known to be length biased in the estimation of average condition in fish (Rennie & Verdon, 2008). To evaluate SL-related bias in the condition index, data from FB and HS were pooled and plotted against SL. The most suitable regression model for this relationship was selected using Akaike’s second order information criterion (AIC_c_; Burnham & Anderson, 2002), where models tested included linear, quadratic, exponential, and cubic distributions, and model parameters and level of significance were calculated. Length-related bias was defined by a coefficient of determination (R^2^) > 0.8, and significant *p*-value in the linear model, which is similar to the method used by studies that examine relative weights using standard weights (Gerow, Hubert & Anderson-Sprecher, 2004).

### Weight-shell length relationships

In the case of length-bias in CI_DW_, DW-SL relationships were developed for *M. truncata* collected from each site in FB and HS. The most suitable regression model for each relationship was selected using AIC_c_. Analyses of Covariance (ANCOVAs), designed to test for interactions between variables, were performed between populations in FB and HS to test for differences in relationships. When an ANCOVA indicated a significant difference in weight-SL relationships between populations, the data were plotted with the 95% confidence intervals (CIs) to identify where the difference occurred. Sites were compared within communities, *i.e.,* sites I1 *vs.* I2 and K1 *vs.* K2, and between communities, *i.e.,* I1 *vs.* K2 and I2 *vs.* K2, restricting the within and between community comparisons to populations that exhibited similar size ranges (*i.e.,* K1 clams did not exceed 47 mm SL and were removed from the between community comparisons).

### Data cleaning and software

A total of 8 outliers, 1 from site I1 (34 mm in SL, ripe female), 4 from site K1 (all<17 mm SL and undifferentiated), and 3 from site K2 (all<24 mm SL and undifferentiated), all with weights outside the normal range of clams collected, were removed from the data set by testing linear regressions of DW *vs.* log SL using the Bonferroni Outlier Test, which detects outliers that have the ability to shift the mean (Doornbos, 1981). One clam collected from Iqaluit was removed from analysis as it was 80 mm, and outside the normal SL range of clams collected. All statistical analyses were performed in R version 3.5.1 for windows (R Core Team, 2018) and the significance level was set to *α* = 0.05. This study was reviewed and approved by Memorial University’s Institutional Animal Care Committee (Project # 18-01-SG).

## Results

### Shell length

Overall, 337 truncate soft-shell clams were examined: 115 male, 131 female, and 91 immature (*i.e.,* undifferentiated (82) and indifferent (9) stages combined). There were more female truncate soft-shell clams collected at sites I1 and K1 while the sex ratios were equal or nearly equal at sites I2 and K2 (Table 1). Analysis indicated the sex of animals examined was independent of site (Chi-squared =3.77, *df* = 3, *p* = 0.29).

**Table 1:** Numbers of immature, female, and male truncate soft-shell clams examined at each site, and the resulting F:M sex ratio.

Community	Site	Immature	Female	Male	Sex Ratio (F:M)
Iqaluit	I1	4	24	13	1.85:1
	I2	9	23	24	0.95:1
Kimmirut	K1	42	15	9	1.67:1
	K2	36	69	69	1: 1
All Sites Combined		91	131	115	1.14:1

**Figure 2:**
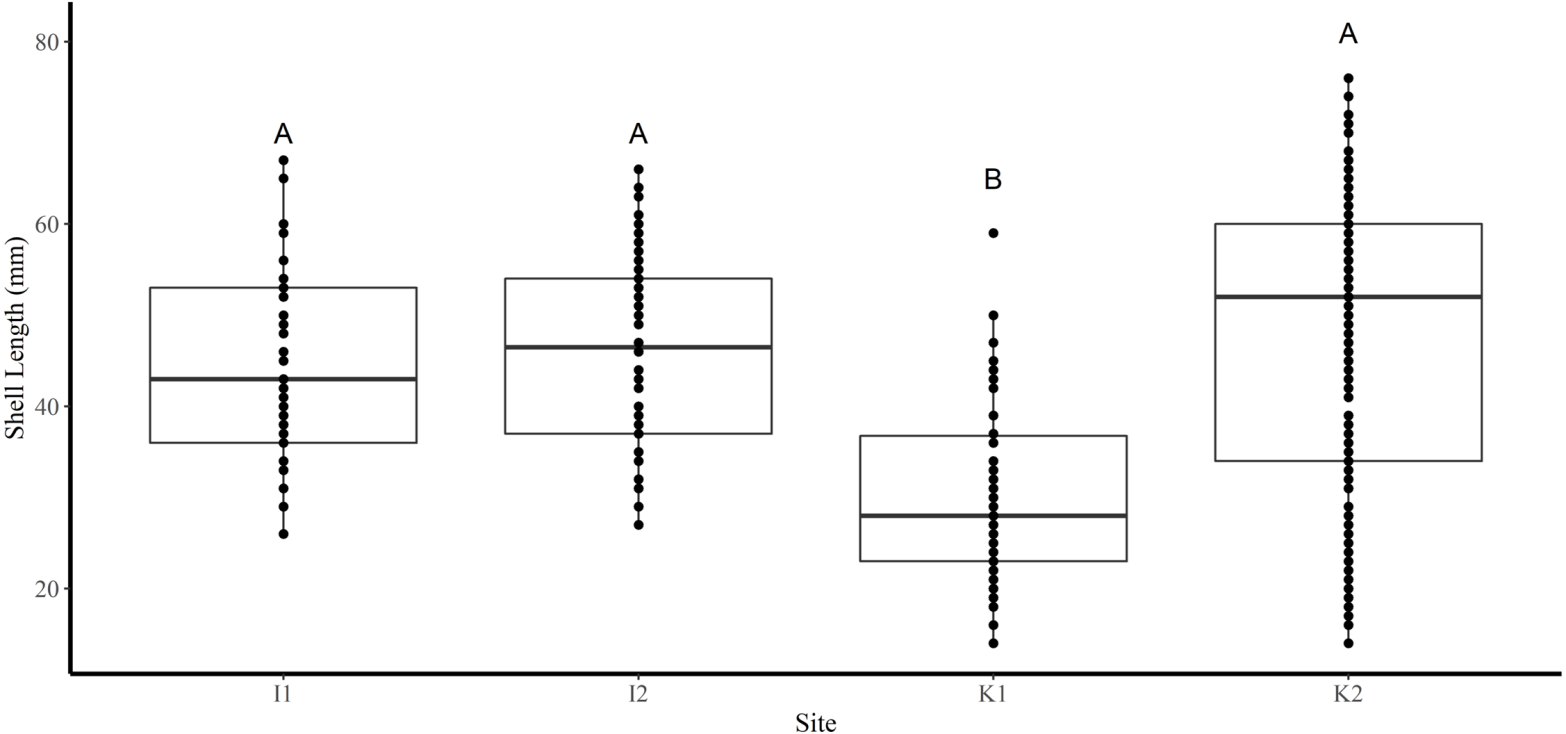
Boxplots of the shell length (mm) of truncate soft-shell clams collected from two sites near Iqaluit (I1 and I2), and two sites near Kimmirut (K1 and K2). Homogenous subsets *via* ANOVA and post-hoc analysis are shown (*i. e.,* A and B). Horizontal lines within the boxes represent the median density. Lower and upper edge of the boxes show the first and third quartiles, respectively. Lower and upper whiskers represent density estimates outside of the interquartile range.

**Figure 3:**
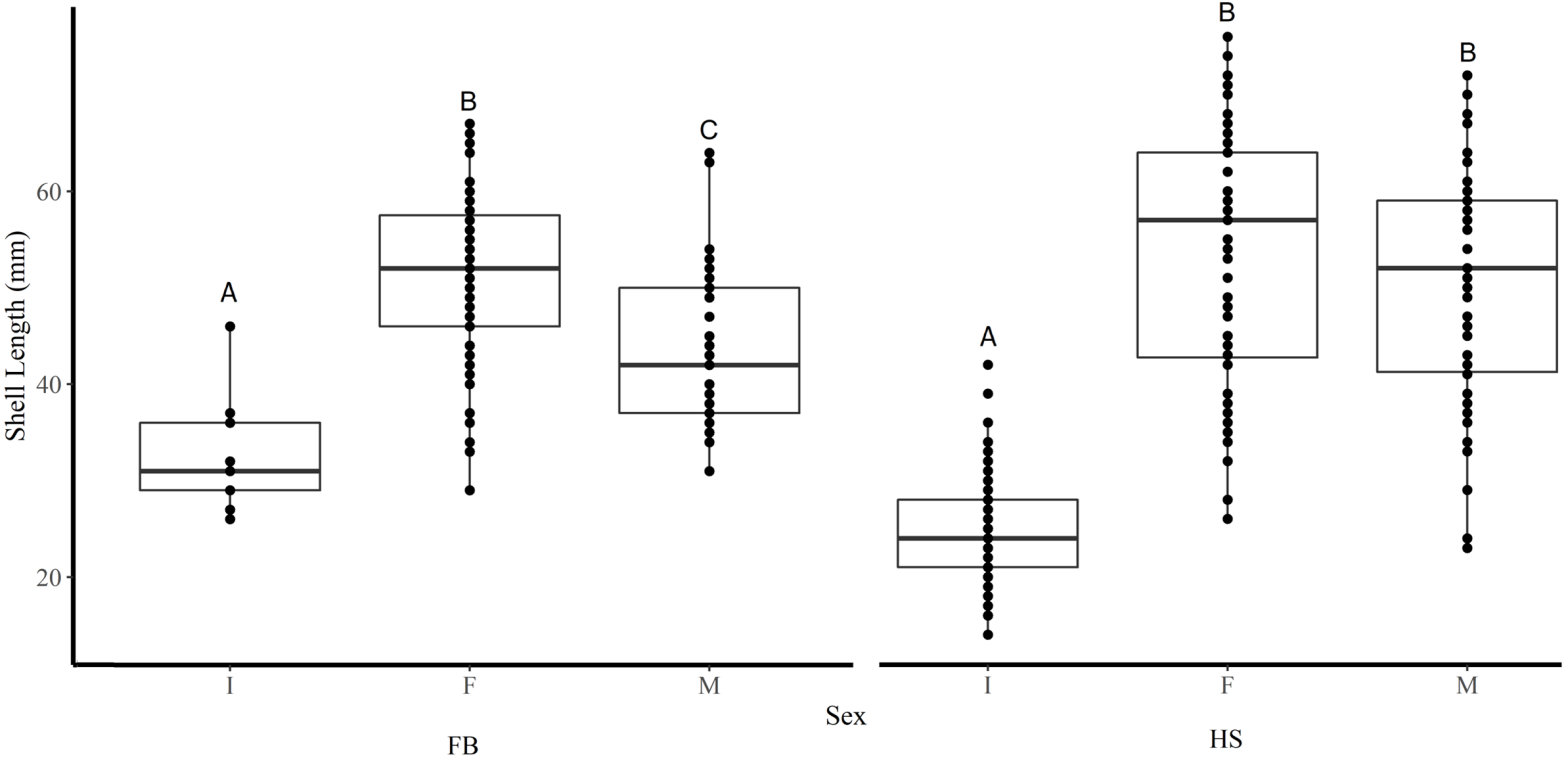
Boxplots of the shell length of Immature (I), Female (F), and Male (M) truncate soft-shell clams collected from Frobisher Bay (FB) and Hudson Strait (HS). Homogenous subsets *via* ANOVA and post-hoc analysis are shown (*i. e.,* A, B, and C). Horizontal lines within the boxes represent the median density. Lower and upper edge of the boxes show the first and third quartiles, respectively. Lower and upper whiskers represent density estimates outside of the interquartile range.

Shell length differed significantly among sites where the average shell length of clams collected at site K1(30 mm ± 1.2 SE) was significantly lower than site K2 (47.8 mm ± 1.2) and both sites in FB (I1 44.5 mm ± 1.6, I2 46.4 mm ± 0.6) (Tukey’s HSD all *p* < 0.001) (Fig. 2). Analysis revealed that the mean SL of immature clams did not differ significantly between FB and HS (32.5 mm ± 1.5, 24.4 mm ± 0.6, respectively) (Tukey’s HSD *p* = 0.09; Fig. 3). However, the mean SL of immature clams from each community was significantly lower than mature males and females from each community (*p* < 0.01). The mean SL of females from FB (50.8 mm ± 1.4) did not differ significantly from females (53 mm ± 1.4) or males (50.6 mm ± 1.3) from HS (*p* = 0.9, *p* = 1.0, respectively). Males from FB (43.6 mm ± 1.4) were significantly smaller than females from FB and males and females from HS (*p* = 0.02, *p* < 0.01, *p* < 0.01, respectively; Fig. 3).

### Stage of gonadal development

In Frobisher Bay, 11.3% of the truncate soft-shell clams examined were classified as undifferentiated, 2.1% were in the indifferent phase of gonadal development, 8.2% were developing, 77.4% were ripe, 1.0% were spawning, and 0% were spent. In the Hudson Strait, 30.5% of clams were undifferentiated, 4.1% indifferent, 30.0% developing, 34.9% ripe, 0.5% spawning, and 0% were spent. Overall, ripe individuals accounted for 89.3% of the mature clams examined from FB and 53.5% of the mature clams from HS.

### Maturity ogive

A logistic regression with a binomial distribution and logit link provided the best fit to both the male and female maturity data based on AICc scores (Table 2). This model is represented by the formula log(p/1-p) = α + βX where p represents the probability of being mature, X is shell length, and α and β are constants.

**Table 2:** Akaike’s information criteria (AICc) values obtained from four different models applied to maturity curves for male and female truncate soft-shell clams. Models in bold font exhibited the lowest AICc value and highest weight.

	Logistic model	AICc	dAICc	df	Weight
Males					
	** Logit**	** −1.2**	** 0.0**	** 2**	** 0.38**
	Probit	−1.3	0.1	2	0.36
	Gompertz	−1.7	0.8	2	0.25
	Weibull	−3.8	8.5	3	<0.01
Females					
	** Logit**	** −1.2**	** 0.0**	** 2**	** 0.39**
	Probit	−1.2	0.2	2	0.36
	Gompertz	−1.5	0.9	2	0.25
	Weibull	−3.8	8.8	3	<0.01

**Figure 4:**
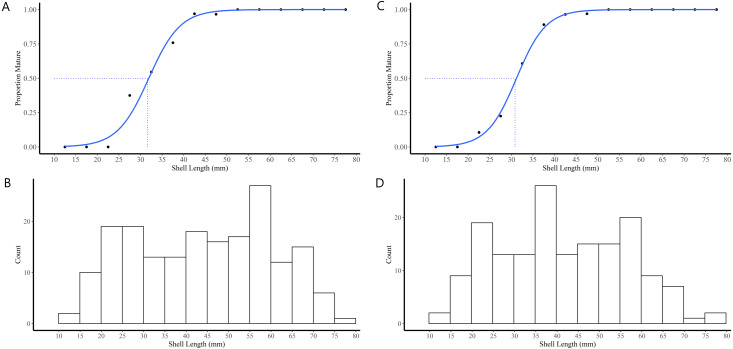
Shell length maturity ogives for female (A) and male (C) truncate soft-shell clams from southern Baffin Island, Nunavut, and histograms of the number of female (B) and male (D) clams examined at each five mm shell length-class. Shell length at 50% maturity is also shown.

The majority (90.1%) of the 91 truncate soft-shell clams classified as immature did not possess gonads while 9.9% possessed gonads in the early stage of differentiation (*i.e.,* indifferent). The immature clams exhibited a range in SL of 11–40 mm. In females, the SL at first attainment of sexual maturity was 28 mm, SL at 50% attainment of sexual maturity (L_50_) was 32 mm, and all females exhibiting a SL ≥42 mm were sexually mature (Fig. 4A). For the males, SL at first attainment of sexual maturity was 23 mm, SL at L_50_ was 31 mm, and all males exhibiting a SL ≥42 mm were sexually mature (Fig. 4C).

### Body condition

The exponential model provided the best fit to the pooled CI_DW_-SL relationship (Table 3). Pooled data across all sites showed significant positive relationships and a high coefficient of determination between CI_DW_ and SL (*n* = 337, *R*^2^ = 0.837, *p* < 2.2^−16^), precluding its utility to compare average condition among the populations studied (Gerow, Hubert & Anderson-Sprecher, 2004; Rennie & Verdon, 2008).

**Table 3:** Akaike’s information criteria (AICc) values obtained from four models applied to dry weight condition index (CI_DW_) versus shell length (SL) relationships for truncate soft-shell clams collected from all four study sites. Model in bold exhibits the lowest AICc value and highest weight.

Model	AICc	dAICc	df	Weight
Linear	2704.5	2387.7	3	<0.001
Quadratic	2705.6	2388.8	4	<0.001
**Exponential**	**316.8**	**0.0**	**3**	**1**
Cubic	2697.4	2380.6	5	<0.001

**Figure 5:**
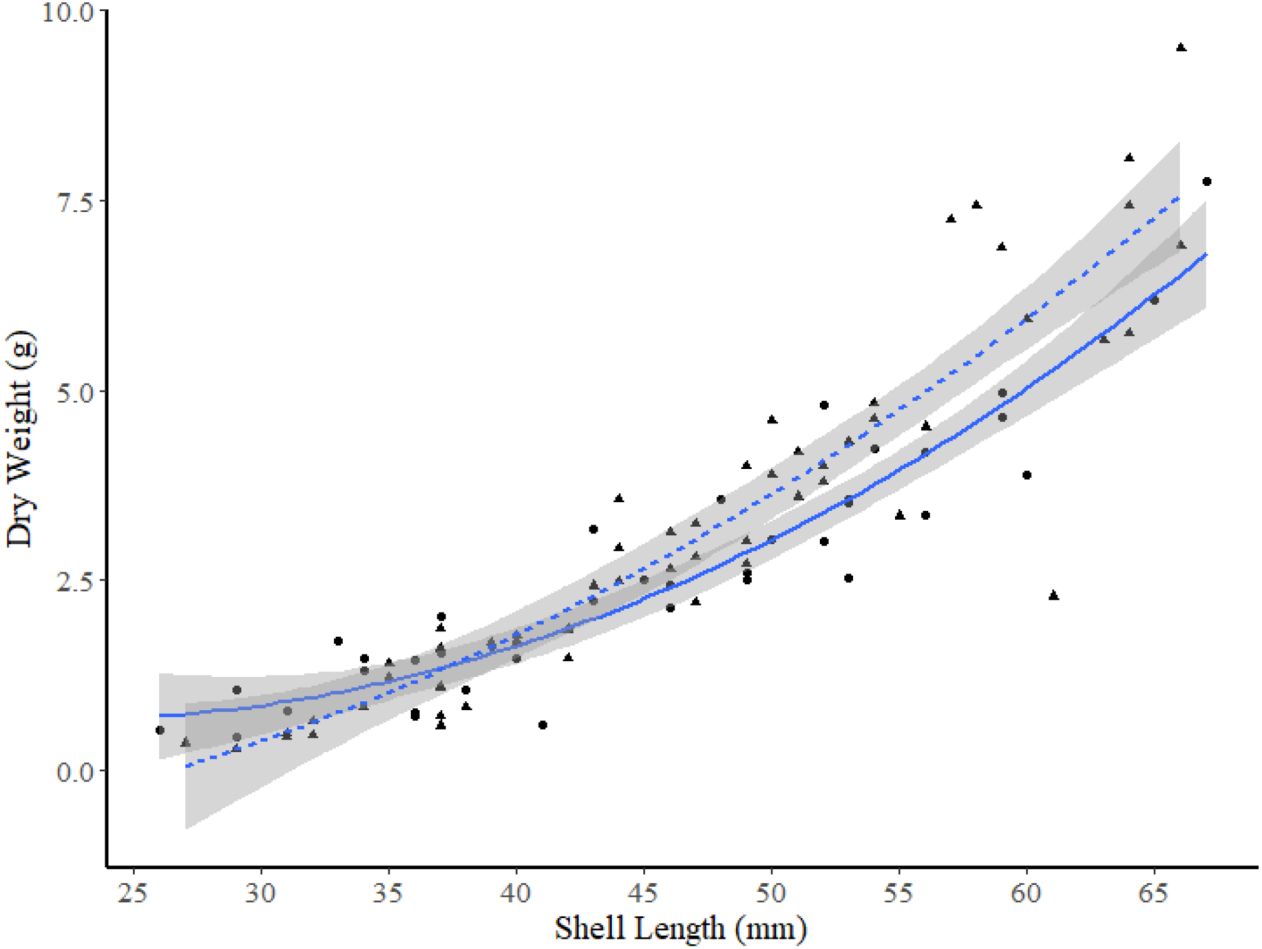
Dry weight *versus* shell length relationship for truncate soft-shell clams from two sites within Frobisher Bay; sites I1 (solid line and circle points) and I2 (dashed line and triangular points). The 95% confidence intervals are shown in grey.

The exponential model was the best model to use for comparisons of the DW-SL relationships between sites within FB and HS (Table 4), and all DW-SL relationships were highly correlated (Table 5). ANCOVA revealed the only significant difference in DW-SL relationships occurred between sites in FB (slope t-value =2.965, *p* = 0.004, intercept t-value = −1.864, *p* = 0.070). Plots of the line of best fit with 95% CIs indicate clams within the 49 to 63 mm SL range from site I2 exhibited a significantly higher dry weight at SL than clams from site I1 (Fig. 5).

**Table 4:** Akaikes information criteria (AICc) values obtained from four models applied to dry weight versus shell length (SL) relationships for each site within Frobisher Bay and Hudson Strait. Model in bold exhibits the lowest AICc value and highest weight.

	Logistic Model		AICc	dAICc		df			Weight	
Site I1	Linear	88.3		144.8		3	<0.001		
	Quadratic	77.1		133.6		4	<0.001		
	**Exponential**	**−56.5**		**0.0**		**3**	**1.0**		
	Cubic	77.6		134.1		5	<0.001		
Site I2	Linear	154.7		243.9		3	<0.001		
	Quadratic	152.7		241.9		4	<0.001		
	**Exponential**	**−89.2**		**0.0**		**3**	**1.0**		
	Cubic	155.3		244.4		5	<0.001		
Site K1	Linear	424.6		492.4		3	<0.001		
	Quadratic	232.8		300.6		4	<0.001		
	**Exponential**	**−67.8**		**0.0**		**3**	**1.0**		
	Cubic	327.1		395.0		5	<0.001		
Site K2	Linear	59.2		113.3		3	<0.001		
	Quadratic	23.6		77.7		4	<0.001		
	**Exponential**	**−54.1**		**0.0**		**3**	**1.0**		
	Cubic	25.9		80.0		5	<0.001		

**Table 5:** Summary of regression constants and correlation coefficients (r^2^) for dry weight versus shell length (SL) relationships obtained for two sites near Iqaluit and two sites near Kimmirut. The number of clams (n) at each site is also shown.

			Regression Constants		
Community	Site	*n*	Slope	Intercept	r^2^
Iqaluit	I1	41	0.13	3.44	0.772
	I2	56	0.09	3.52	0.801
Kimmirut	K1	64	0.35	3.09	0.720
	K2	72	0.32	3.10	0.744

**Figure 6:**
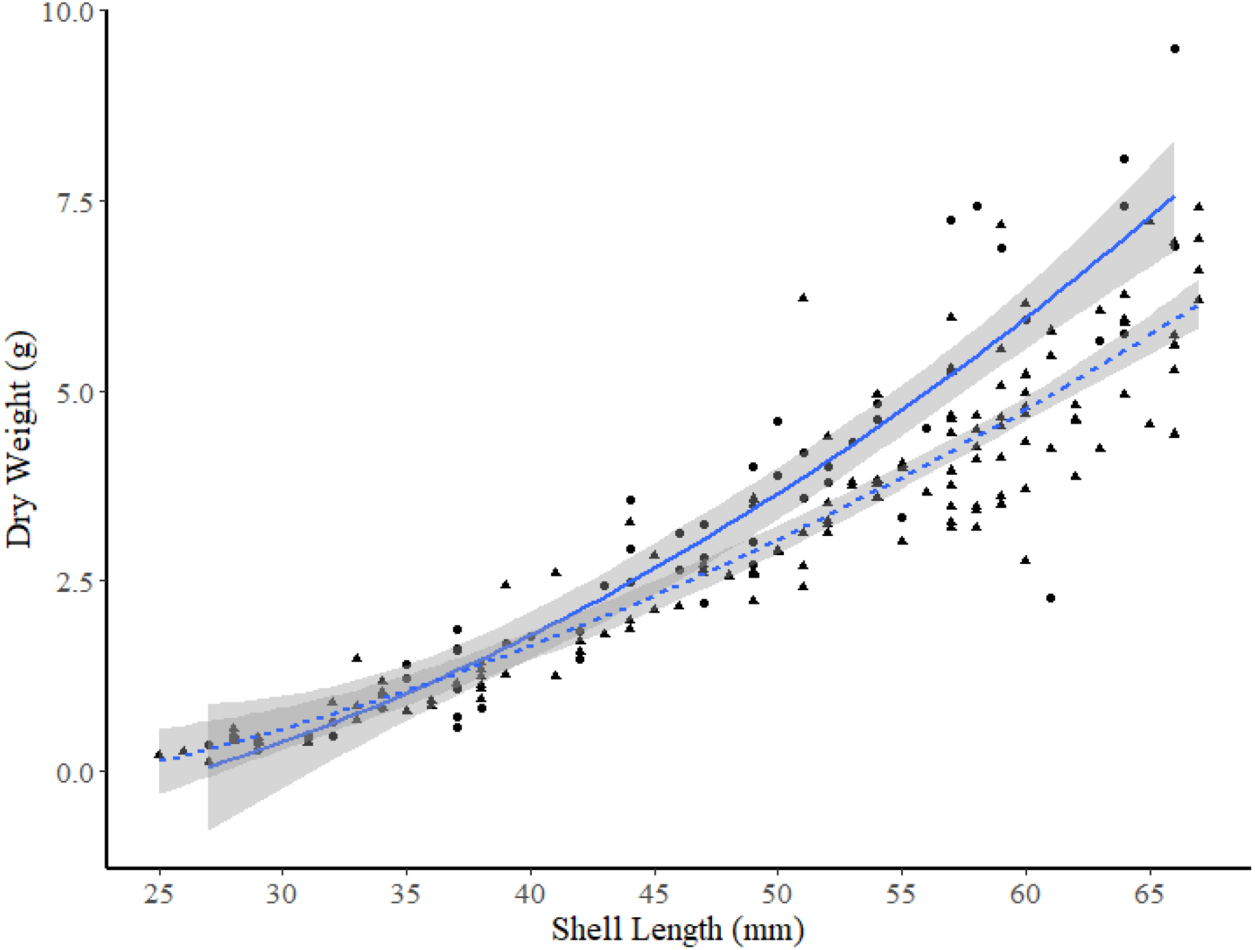
Dry weight *versus* shell length relationships for truncate soft-shell clams from two sites: one within Frobisher Bay (I2; solid line and circle points), and one within Hudson Strait (K2; dashed line and triangular points). 95% confidence intervals are shown in grey.

Among the two FB sites and site K2, ANCOVA analysis found no significant differences between DW-SL relationships between site I1 and K2 (slope t-value =−0.243, *p* = 0.808, intercept t-value = −0.142, *p* = 0.887). However, subsequent pair-wise comparisons between the two sites in FB and site K2 revealed that the DW-SL relationships of sites I2 and K2 differed significantly (Estimate = 0.600, S.E. = 0.120, *df* = 224, t-ratio = 4.99, *p* < 0.001). Figure 6 shows that clams ≥49 mm SL from site I2 had higher DW than similar sized clams from site K2.

## Discussion

According to the maturity ogive which was constructed based on samples taken in late summer, the size at which 50% of the *M. truncata* population of southern Baffin Island is sexually mature is 31 mm in males and 32 mm in females. This size is small in comparison to the size at which *M. arenaria*, a sister species to *M. truncata,* is sexually mature in other parts of Canada, such as in the Gulf of St. Lawrence at 41 mm (Department Fisheries and Oceans Canada (DFO), 1996). This could be due to differences between species, or that clams occurring in colder waters have slower growth rates and mature at a smaller size compared to southern populations (Weymouth, McMillin & Rich, 1931). It should be noted that *M. truncata* have truncated shells, which may also influence comparisons of the size at maturity between *M. truncata* and *M. arenaria*. It is conceivable that the significant difference in shell length between males and females from FB and of FB males compared to both sexes from HS could have affected maturity ogives. However, given that *M. arenaria* does not show sex-specific differences in growth rates (Brousseau, 1979), it would seem unlikely that differences in SL would influence our results. Further, FB males accounted for only 32% of the males examined and their size distribution was within the range of males and females from all sites. These observations lead us to conclude shell length differences between sexes and populations had no influence on the maturity ogives.

Most of the mature clams collected in FB in late August and just over half of the mature clams collected in HS two weeks later in mid-September were in the ripe stage of gonadal development. *M. arenaria* in the northwest Atlantic commence gametogenesis in late winter or early spring and spawn from June to September, sometimes with a second fall spawning event taking place (reviewed by Christian, Grant & Meade, 2010). Spawning in bivalves is thought to be triggered by increasing water temperatures, such as in the spring (Chicharo & Chicharo, 2000), and fluctuations in chlorophyll *a* concentration, where phytoplankton blooms provide food for larvae (Brandner et al., 2017; Chicharo & Chicharo, 2000). The spawning period of *M. truncata* has been observed to occur in April in the North Sea (Amaro, Duineveld & Tyler, 2005), with larvae observed in the water column of the White Sea from June to August, peaking in July (Günther & Fedyakov, 2000). Brandner et al. (2017) used D-shaped larvae abundance to infer the spawning time of various bivalve species in Western Svalbard, as D-shaped larvae are the first larval stage to occur post-spawning (Sullivan, 1948). They observed D-shaped *M. truncata* larvae in May with maximum chlorophyll *a* concentration, and a second cohort in September when the chlorophyll *a* concentration was low (Brandner et al., 2017). To summarize, past research has shown that *M. truncata* spawn in early spring, occasionally with a second fall spawning event.

Another factor that may help to infer the spawning period of *M. truncata* is phytoplankton abundance as larval *Mya* sp. filter feed on surrounding microplankton, such as autotrophic phytoplankton and cyanobacteria (Raby et al., 1997). During ice-covered months in FB, ice algae occupy the underside of the ice and are of higher concentration than phytoplankton in the water beneath, and algae mix with phytoplankton to produce a maximum chlorophyll *a* concentration in the summer once ice has melted (Hsiao, 1992). In HS, chlorophyll *a* concentration due to phytoplankton production is highest in the summer for similar reasons (Harvey, Therriault & Simard, 1997). Around southern Baffin Island, ice cover occurred from the end of November 2017 to mid-July 2018, and ice cover returned towards the end of November 2018 (Canadian Ice Service, 2018). Therefore, *M. truncata* collected from southern Baffin Island during this study may have spawned from late September to early November to align with chlorophyll *a* concentration for feeding larvae before ice cover returned. Conversely, *M. truncata* collected during this study may have spawned in spring-summer to align with the maximum summer chlorophyll *a* concentration and had already commenced gametogenesis for next spring-summer spawning. It is unclear whether *M. truncata* collected during this study had spawned in the spring-summer and were preparing for a second fall spawning event as observed by Brandner et al. (2017). Spawning is an energy-demanding process for clams, including *Mya* sp. (Beninger & Lucas, 1984; Darriba, Juan & Guerra, 2005; Gauthier-Clerc et al., 2002), and energy demands for spawning and subsequently post-spawning condition may dictate whether a second spawning event takes place in a year. To fully understand the spawning season of *M. truncata* in Canadian Arctic waters, it will be important to seasonally monitor stages of gonadal development. Future studies should also consider whether variation in ice cover from year-to-year and differences in phytoplankton species composition and production that may arise with climate change can influence the spawning season. It is noteworthy, that size and condition were found to be more important than age for determining spawning in *M. arenaria*, and so the age of the populations examined in this study is hypothesized to have no effect on spawning potential (Newell & Hidu, 1986). This study provides a baseline to monitor potential changes in aspects of the life history of truncate soft-shell clams of southern Baffin Island with climate change-induced environmental fluctuations.

Many fisheries and aquaculture operations use body condition to infer the health of a population, including bivalves (Lucas & Beninger, 1985). In the past, mollusc studies using condition factor indices have either not checked for length-bias in data (*e.g.*, Pillay, Branch & Forbes, 2007), constrained size ranges to avoid length bias altogether (*e.g.*, Ellis et al., 2002), or used weight to length relationships instead of condition factor indices (*e.g.*, Eckblad, 1971). In the current study, strong relationships were identified between CI_DW_ and SL, indicating length-related bias in this calculated condition index. Therefore, the present study assessed body condition between sites using DW to SL relationships. The use of standard weight condition indices was not a viable option for the present study as *M. truncata* length and weight data is not available for the geographic extent of the species. This study only examined clams from four sites, but will contribute length and weight data for a future database for eastern Nunavut to derive standard weights for *M. truncata* using relative weight condition indices. Since clams in higher condition are defined as having a greater mass than their counterparts at the same length, weight-SL relationships allow for condition analyses when there is not enough data to examine relative weights to standard weights. Future studies may also want to consider the use of condition indices outlined by Higgins (1938) among others (*e.g.*, Crosby & Gale, 1990) that use shell volume instead of shell length for bivalves, as shell volume may help to eliminate length-bias concerns.

For the DW- SL relationship observed between FB sites, a significant difference emerged at a SL of 52 mm. At this size, 100% of male and female clams were mature. Therefore, it is possible that body condition may be affected by stage of gonadal maturity. As gametogenesis involves building gametes from energy reserves, when there is limited or no food, the condition of clams post-spawning would be lower than during gametogenesis (Gauthier-Clerc et al., 2002). Therefore, in the future, it would be beneficial to account for how body condition may vary with time as clams cycle through gonadal development. Nevertheless, the body condition of bivalves can be the result of a variety of factors, such as current velocity, phytoplankton abundance, sediment suspension, or sewage effluent and heavy metal contamination that could lower body condition (Filgueira et al., 2014; Jorgensen, 1996; Page et al., 1984; Nobles & Zhang, 2015). These factors may have contributed to differences in condition observed on small and large spatial scales, along with other anthropogenic factors such as differences in fishing pressure and subsequent density (*i.e.,* site I1 and K1 are not as heavily harvested as I2 and K2). Spatial differences in body condition may also infer differences in reproductive potential, as body condition in bivalves at the ripe stage of gonadal development, right before spawning, tends to be higher than at other periods of gonadal development (*e.g.*, Adjei-Boateng & Wilson, 2013; Etim, 1996), and higher body condition infers higher reproductive output in bivalves (Jokela, 1996). These hypotheses should be tested in future studies to thoroughly understand the reproductive potential of populations of *M. truncata* of southern Baffin Island as it relates to biological condition.

## Conclusion

This study provides a first estimate of the size at sexual maturity for *M. truncata* populations in the southern region of Baffin Island and demonstrates a method to assess and compare body condition of populations in the absence of sufficient data to derive standard weight equations. This is the first study to investigate the stage of gonadal development in *M. truncata* in the Baffin Island region. Because our analysis was limited to a single point-in-time collection, it was not possible to determine the spawning cycle or the potential for two spawning events per year. Given the geographic location of our study and limited ice-free season for phytoplankton production, we suspect that the high proportion of ripe clams collected in August-September is indicative of a late summer-autumnal development of the gonad and gametes while food availability is high. Strong length-bias was identified in condition indices for these populations, which led to the use of dry body weight to shell length relationships to compare individual condition among populations. This study provides baseline information to monitor how Arctic *M. truncata* will be affected by potentially life history-altering climate change effects for conservation and management purposes and is expected to help future community-based resource harvest plans for recreational fishery development that has the potential to provide significant benefits in terms of revenues and job creation.

## Supplemental Information

10.7717/peerj.13231/supp-1 Supplemental Information 1 Raw data Shell length measurements, dry body weight measurements, and calculated condition indices per described methods from clams collected at all four described study sites. This data was used for statistical analyses performed for this study.

## Additional Information and Declarations

### Field Study Permissions

The following information was supplied relating to field study approvals (i.e., approving body and any reference numbers): Memorial University’s Institutional Animal Care Committee (Project # 18-01-SG).

### Data Availability

The following information was supplied regarding data availability: The raw data is available in the Supplemental File.
